# Unveiling the Synergistic Effects of Melatonin, Arginine, and Nano‐Chelated Zn‐Fe on Enhancing Fruit Quality in Apricot (*Prunus armeniaca*)

**DOI:** 10.1002/fsn3.70559

**Published:** 2025-07-22

**Authors:** Raghad Adnan Ali AL‐Qady, Wasan Waleed Ahmad, Waad S. Faizy, Mustafa Natheer Mustafa, Borzou Yousefi, Heidar Meftahizade

**Affiliations:** ^1^ Department of Biology, College of Education for Pure Sciences Al‐Hamdaniya University Al‐Hamdaniya Iraq; ^2^ Department of Plant Production Techniques, Agricultural Technical College Northern Technical University Mosul Iraq; ^3^ Department of Medicinal Plants, Kermanshah Agricultural and Natural Resorces Research and Education Center AREEO Tehran Iran; ^4^ Department of Horticultural Sciences, Faculty of Agriculture & Natural Resources Ardakan University Ardakan Iran

**Keywords:** amino acids, fruit firmness, nutritious, soluble sugars, vitamin

## Abstract

Apricot ( *Prunus armeniaca* ) is a delicious and highly nutritious fruit, rich in organic acids, various vitamins, sugars, proteins, and minerals, and possesses multiple medicinal properties. To enhance fruit yield and quality while reducing the use of chemical fertilizers, an experiment was conducted on the basis of a randomized complete block design with three replications in the north of Dohuk, Iraq during 2023. Apricot trees were foliar‐sprayed with 28 different combination levels of arginine, melatonin, and nano‐chelated zinc–iron. The results showed that most treatments led to improvements in both quantitative and qualitative traits of apricot fruits compared to control. Among them, the application of arginine 150 ppm + melatonin 400 μmol/L + Zn‐Fe nano‐chelate 2 and 3 g/L resulted in the most significant increases in fruit firmness (21.14% and 21.93%), fruit weight (35.60% and 35.35%), fruit length (25.77% and 20.26%), fruit yield (27.11% and 26.95%), the contents of vitamin C (20.73% and 20.39%), soluble sugars (33.51% and 33.58%), and total soluble solids (17.60% and 17.72%) compared to control. These treatments also improved the concentrations of calcium (49.26% and 47.84%), magnesium (20.97% and 26.36%), potassium (58.40% and 67.43%), zinc (11.19% and 19.26%) and iron (9.46% and 11.07%) in the fruit, while reducing total acidity. In addition, they enhanced leaf area and chlorophyll content (SPAD index). Ultimately, these two treatment combinations are recommended for improving both the quantity and quality of apricot production.

## Graphical abstract

Highest levels of mineral materials and vitamins were observed in arginine (150 ppm) + melatonin (400 μmol/L) + Nano‐chelated Zn‐Fe at either 2 or 3 g/L. T27 and T28 resulted in the highest fruit yield, total soluble solids, fruit set, and firmness, along with reduced acidity. 150 ppm + 400 μmol/L + 2 g/L and 150 ppm + 400 μmol/L + 3 g/L are recommended as the most effective for enhancing yield and fruit quality.

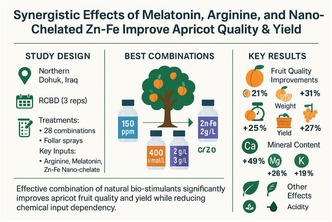

## 1 Introduction

Although chemical fertilizers have a positive and direct effect on increasing crop production, on the other hand, they have a negative impact on the environment and human health. The excessive and long‐term application of chemical fertilizers causes soil compaction, degradation and acidification, and a decrease in bacterial diversity, resulting ultimately in a decrease in soil organic matter and fertility (Bisht and Chauhan 2020; Tadayon et al. 2025; Wang, Wu, et al. 2023). Nutrients and human health‐promoting compounds can be enriched in crops and horticultural products through optimizing pre‐harvest management practices (Guardiola‐Márquez et al. 2022; Wang, Li, et al. 2023). The application of nano‐materials, biofertilizers, and biostimulants is a promising approach because of plant nutrition efficiency, eco‐friendliness, and sustainability (Dasgan et al. 2022; Hussein et al. 2024; Suchithra et al. 2022). Fertilizers affect vegetative growth, flowering, fruit set, and fruit retention and have a marked effect on fruit quality and yield (Abdelmigid et al. 2022). Although the use of chemical fertilizers increases fruit yield, it also reduces fruit quality (Almadiy et al. 2023; Wan et al. 2021). Recent research suggests that biostimulants improve crop yield and quality, shrink the application of fertilizers, upsurge water‐use efficiency, and boost plants tolerance to stress (Meena et al. 2025). The use of amino acids as biostimulants in sustainable production has recently been considered. Amino acids are organic molecules that contain N, C, H, and O_2_ (Buchanan et al. 2015).

Arginine has been identified as essential in nitrogen storage. It is effective for transporting in plants because of the high nitrogen/carbon ratio (Chen et al. 2022). Arginine can also undergo decarboxylation by arginine decarboxylase (ADC) and be converted to guanidine butylamine and then putrescine and other polyamines (Patel et al. 2017). These polyamines stimulate plant growth and production. Some reports indicated that arginine solely or in combination has increased fruit yield and quality of different fruits tree. For example: the foliar application of the arginine in Guava Trees improved markedly the fruit set percentage, fruit yield, fruit firmness, fruit content of total soluble solids (TSS %), vitamin C (VC), and total sugars as well as the leaf potassium content compared to control (Almutairi et al. 2022). Also, 400 μM arginine moderated pH in cherry fruits, whereas it has increased soluble solids, vitamin C, and the firmness of fruits (Pakkish and Mohammadrezakhani 2022).

Melatonin is widely involved in plant biological and physiological processes including photosynthesis, plant growth, flowering, seed and pollen germination, fruit production, rhizogenesis, and senescence (Himanshu et al. 2024; Saroj et al. 2023; Zhu et al. 2024). Melatonin triggers signaling cascades of Mitogen‐Activated Protein Kinase (MAPK) and stimulates antioxidant enzymes including superoxide dismutase, catalase, and peroxidase, which scavenge reactive oxygen species (ROS) such as hydroxyl radicals, superoxide anions, and hydrogen peroxide (Kołodziejczyk and Kaźmierczak 2024). Melatonin improved photosynthetic efficiency by reducing chlorophyll degradation and alleviating the damage to the integrity of the thylakoid membrane by increasing the expression levels of electron‐transport‐related proteins, and raises the efficiency of photosystems I and II through increasing light absorption and energy transfer inside the chloroplasts (Amin et al. 2022; Hussain et al. 2024; Shahani et al. 2023; Sharma et al. 2020; Yan et al. 2023) (Lin et al. 2022; Yang et al. 2022).

Zinc and iron participate in the lipids, proteins, and carbohydrates metabolism, antioxidant activity, DNA replication, and transcription (Fatemi et al. 2020; Guardiola‐Márquez et al. 2023). The Zn and Fe Nano‐scale, because of their extremely small size and large surface‐to‐volume ratio and unique physicochemical properties, have enhanced reactivity, adsorption capacity, and functionalization properties compared to their bulk counterparts, improving their uptake, transport, and efficiency (Hussein et al. 2024). Foliar spraying of ZnO‐NPs has enhanced tomato height, early flowering, fruit yields, as well as lycopene content (Ahmed et al. 2021). Foliar application of Nano‐Zn at 200 μg g^−1^ improved vegetative growth, fruit set (16.9%), yield (48.3%), and metabolic content of strawberry fruits compared to control and bulk Zn nutrient analogues (Saini et al. 2021).

Apricot ( *Prunus armeniaca* ) is a stone fruit that is highly appreciated by consumers for its pleasant taste and flavor; its nutritional characteristics; and its high concentrations of bioactive compounds with antioxidant activity including phenolics and vitamins. (Fan et al. 2018). It is rich in vitamins including A, C, K, E, and B; crude fiber; crude fat; sugars; proteins; minerals; and organic acids (Al‐Saif et al. 2023; Al‐Soufi et al. 2022). The presence of antioxidants in apricot has been shown to have a variety of health benefits, including healthy vision, improved circulation, healthiness of the heart, and improved immune system functions; additionally, the qualities of apricot fruits are subject to change on the basis of agri‐management practices, including firmness, sugar quality, and uneven fruit ripening, which can all feature in apricots and affect their nutritional configurations (Al‐Soufi et al. 2022).

Although previous studies have investigated the effects of arginine, the hormone melatonin, and Zn‐Fe Nano‐chelates separately on a number of fruit trees and many crops, the combined use of these three compounds in apricots and other crops has not been tested so far. Therefore, we applied multiple combination treatments of arginine, melatonin, and Zn‐Fe Nano‐chelate to improve the nutritional quality of the apricot fruit while increasing the yield of the apricot crop.

## 2 Materials and Methods

### 2.1 Experimental Design, Plant Material, and Field Treatments

This research was carried out in a randomized complete block design (RCBD) at the experimental site in a private orchard of 12‐year‐old trees in the Bagherat area, north of Dohuk, Iraq (longitude 43.09 east, latitude 36.57 north, elevation 881 m above sea level). For each replicate, 10 fruits were randomly collected from the experimental trees for quality assessments. Uniform apricot trees (Canino cultivar) received the same agricultural practices and were subjected to different combined doses of Arginine + Melatonin + Zn‐Fe Nanochealate (T1–T28) as follows: T1 = 0 ppm + 0 μmol/L + 0 g/L, T2 = 60 ppm + 100 μmol/L + 1 g/L, T3 = 60 ppm + 100 μmol/L + 2 g/L, T4 = 60 ppm + 100 μmol/L + 3 g/L, T5 = 60 ppm + 200 μmol/L + 1 g/L, T6 = 60 ppm + 200 μmol/L + 2 g/L, T7 = 60 ppm + 200 μmol/L + 3 g/L, T8 = 60 ppm + 400 μmol/L + 1 g/L, T9 = 60 ppm + 400 μmol/L + 2 g/L, T10 = 60 ppm + 400 μmol/L + 3 g/L, T11 = 100 ppm + 100 μmol/L + 1 g/L, T12 = 100 ppm + 100 μmol/L + 2 g/L, T13 = 100 ppm + 100 μmol/L + 3 g/L, T14 = 100 ppm + 200 μmol/L + 1 g/L, T15 = 100 ppm + 200 μmol/L + 2 g/L, T16 = 100 ppm + 200 μmol/L + 3 g/L, T17 = 100 ppm + 400 μmol/L + 1 g/L, T18 = 100 ppm + 400 μmol/L + 1 g/L, T19 = 100 ppm + 400 μmol/L + 3 g/L, T20 = 150 ppm + 100 μmol/L + 1 g/L, T21 = 150 ppm + 100 μmol/L + 2 g/L, T22 = 150 ppm + 100 μmol/L + 3 g/L, T23 = 150 ppm + 200 μmol/L + 1 g/L, T24 = 150 ppm + 200 μmol/L + 2 g/L, T25 = 150 ppm + 200 μmol/L + 3 g/L, T26 = 150 ppm + 400 μmol/L + 1 g/L, T27 = 150 ppm + 400 μmol/L + 2 g/L, T28 = 150 ppm + 400 μmol/L + 3 g/L.

The foliar spraying was carried out in a standardized volume and coverage after irrigation. The foliar applications were made using a back‐held sprayer in the early morning. Solutions were prepared by dissolving the allocated amount in the required distilled water. Tween‐20 was added to treatments to facilitate the absorption of treatments. Foliar sprays of all treatments were carried out three times; the first on the 10 of April (at the blossoming stage), the second one on the (15 June), and the third one on the 20 July (fruit set completion stage).

### 2.2 Meteorological Information

**FIGURE 1:**
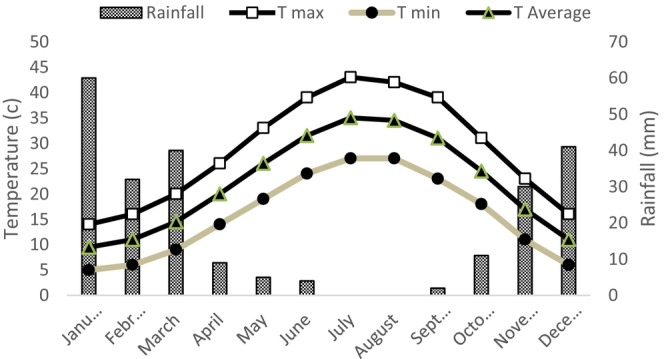
The thermo‐pluviometric trend diagram at the research field (north of Dohuk Iraq) during 2023.

The data of air temperature and precipitation during the trial period were recorded by means of a meteorological data logger located close to the study site (Figure 1). During the trial periods, the minimum temperature was 5°C in March (only 1 day) and a maximum of 43°C in June. Rainfall was 60 mm, of which the maximum occurred in January.

### 2.3 Fruit Yield and Fruit Quality Assessments

The apricot fruits were harvested at optimum maturity. The number of fruits per tree was counted. Average fruit weight (g) using a digital balance and fruit length (cm) using a digital vernier caliper for 10 fruits in each replicate were measured. The average yield/tree (kg) was calculated by multiplying the average fruit weight by the number of fruits/trees. Leaf area (cm^2^): It was measured using a leaf area meter (LI‐3000C, LI‐COR Biosciences); SPAD value: chlorophyll content was estimated using a SPAD‐502 chlorophyll meter (Konica Minolta, Japan); fruit Set (%): it was calculated by the formula: (Number of fruits/Number of flowers) × 100; fruit weight and length: 10 fruits per tree were randomly selected. Weight was measured with a precision scale (0.01 g); length was measured using a digital caliper (Mitutoyo, Japan), fruit firmness (N) was measured using a penetrometer (FT‐327, Effigy, Italy), total soluble solids (TSS) were determined using a digital refractometer (Atago PAL‐1, Japan) and expressed as°Brix; total acidity was determined by titration with 0.1 N NaOH and expressed as % malic acid, following AOAC method 942.15. Vitamin C content (mg/100 g FW) was measured by titration with 2,6‐dichlorophenolindophenol, as described in AOAC method 967.21.

A representative juice sample was taken from each replicate to evaluate chemical properties. The total acidity percentage was determined in accordance with A.O.A.C. (1995). The total soluble solids concentration (Brix%) was measured with a digital refractometer. Vitamin C content was measured as mg/L ascorbic acid/100 mL juice by titration against 2,6‐dichlorophenol‐indophenol (A.O.A.C. 1995).

### 2.4 Determination of the Mineral Elements in Fruit

The collected samples of apricot fruits from each treatment were dried at 35°C and then digested using the wet digestion procedure (H_2_SO_4_–H_2_O_2_) (Parkinson and Allen 1975). The concentrations of K were measured using a flame photometer. The concentrations of Zn, Cu, and Mg were measured by an atomic absorption spectrophotometer. Fe content was measured spectrophotometrically by the phenanthroline method, according to Parkinson and Allen (1975).

### 2.5 Statistical Analysis

The collected data were statistically analyzed using IBM SPSS statistics software (ver. 26), Minitab software (ver. 16), and R‐studio software (ver. i386, 3.2.2). The Multiple Duncan test at the 0.05 level was used to compare means. The graphs designed using Excel software (2010), Minitab software (ver. 16), and R‐studio software (ver. i386, 3.2.2).

## 3 Results

The effect of treatments on the leaf area, SPAD, fruit set, fruit yield/tree, fruit weight, fruit length, fruit firmness, TSS, total acidity, vitamin C, total sugar, and the contents of Ca, Mg, K, and Zn was significant at the level of 1%.

### 3.1 Leaf Area and Photosynthesis

#### 3.1.1 Leaf Area

**FIGURE 2:**
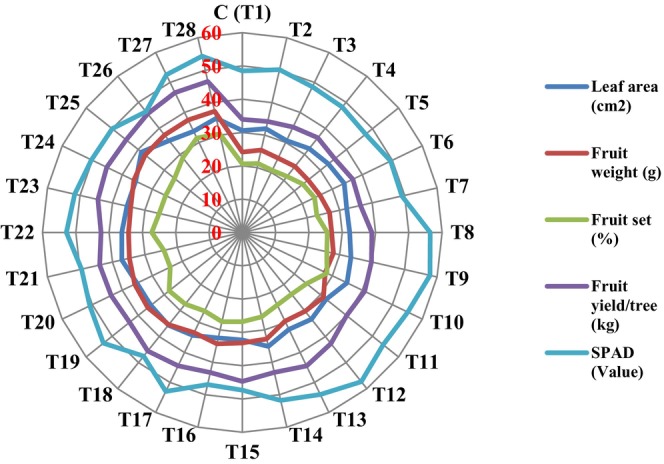
Means comparison (Duncan's Test: *p* < 0.05) leaf area, fruit weight, fruit set, fruit yield/tree, and SPAD value of Apricot under different arginine + melatonin + Zn‐Fe nano‐chelate treatments.

Overall, plants treated with T10, T14, and T17 to T28 showed significantly higher leaf areas than control trees and those treated with other treatments. The lowest leaf area (30.62 cm^2^) was recorded in the control trees, with no significant difference from plants treated with T2 to 5, T7, T8, T11, T13, and T16 (Figure 1). The highest leaf area (38.73 cm^2^) was observed in trees treated with 150 ppm arginine + 200 μmol/L melatonin + 3 g/L Zn‐Fe Nano‐chelate (T25), which was not significantly different from those treated with T21 (37.13 cm^2^). Application of 150 ppm arginine + 200 μmol/L melatonin + 3 g/L Zn‐Fe Nano‐chelate (T25) caused an increase in leaf area by 20.94% compared to the control (Figure 2).

#### 3.1.2 Chlorophyll Index (SPAD)

The highest chlorophyll index (57.83) was measured in plants treated with arginine 60 ppm + melatonin 400 μmol/L + Zn‐Fe Nano‐chelate 2 g/L (T9), with no significant difference from treatments T8, T10–13, T17, T19, and T22 (Figure 2). Spraying trees with treatment T9 caused an increase of 16.13% in SPAD value compared to control, whereas spraying with T28 caused a 10.68% increase. The lowest SPAD value (46.87) observed in plants treated with Argeinine 100 ppm + melatonin 200 μmol/L + Zn‐Fe Nano‐chelate 3 g/L (T16), not significantly different from the control plants and those treated with T5, T15, and T18 (Figure 1).

### 3.2 Fruit Appearance, Physiological, and Yield Traits

Fruit features in terms of the fruit weight, fruit length, and fruit firmness were significantly increased with the application of arginine + melatonin + Zn‐Fe Nano‐chelate compared with those of unsprayed plants. We noticed especially that spraying with arginine 150 ppm + melatonin 400 μmol/L + Zn‐Fe Nano‐chelate 2 g/L and arginine 150 ppm + melatonin 400 μmol/L + Zn‐Fe Nano‐chelate 3 g/L (T27 and T28) created significantly different results from the application of some other treatments.

#### 3.2.1 Fruit Set

The highest fruit set (31.50%) was recorded in plants treated with arginine 150 ppm + melatonin 400 μmol/L + Zn‐Fe Nano‐chelate 2 g/L (T27), followed by T28 (30.37%), both significantly different from others (Figure 1). Spraying with arginine 150 ppm + melatonin 400 μmol/L + Zn‐Fe Nano‐chelate 2 g/L and arginine 150 ppm + melatonin 400 μmol/L + Zn‐Fe Nano‐chelate 3 g/L, respectively, increased fruit set by 34.58% and 32.15% compared to the control. The lowest fruit set (20.61%) observed in the control, not significantly different from plants treated with T2 and T4 (Figure 2).

#### 3.2.2 Fruit Yield/Tree

The highest fruit yield (46.60 kg) was obtained from plants treated with T27, followed by T28 (46.50 kg), which was not significantly different from T13 and T17–T26 (except T19 and T22) (Figure 2). Application of arginine 150 ppm + melatonin 400 μmol/L + Zn‐Fe Nano‐chelate 2 g/L and arginine 150 ppm + melatonin 400 μmol/L + Zn‐Fe Nano‐chelate 3 g/L (T27 and T28) increased fruit yield by 27.11% and 26.95%, respectively, compared to control (Figure 2). The lowest fruit yield (33.97 kg) was obtained from the control plants, not significantly different from those treated with T2–T7.

#### 3.2.3 Fruit Weight

The highest fruit weight (average 37.47 g) was recorded in plants treated with 150 ppm arginine + 400 μmol/L melatonin + 2 g/L Zn‐Fe Nano‐chelate (T27), which was not significantly different from treatments T18–T26 and T28 (Figure 2). Treatments with 150 ppm arginine + 400 μmol/L melatonin + Zn‐Fe nano‐chelate at 2 g/L and 3 g/L (T27 and T28) increased fruit weight by 35.60% and 35.35%, respectively. The lowest fruit weight (24.32 g) was observed in the control group, which did not differ significantly from treatments T2–T8 (Figure 2).

#### 3.2.4 Fruit Length

**FIGURE 3:**
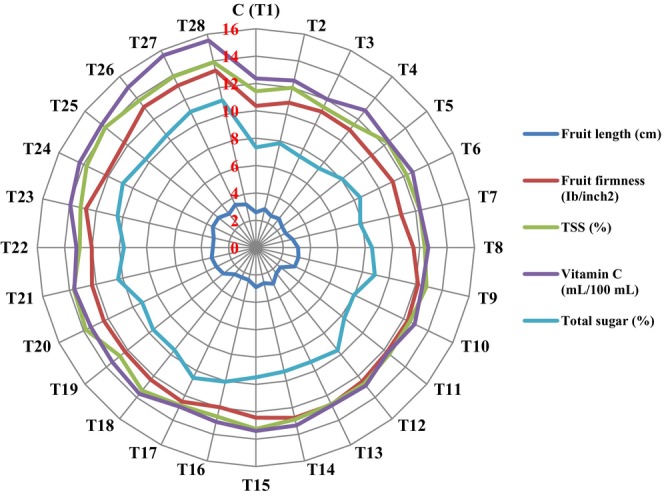
Means comparison (Duncan's Test: *p* < 0.05) fruit length, fruit firmness, TSS, vitamin C, and total sugar of apricot fruit under different arginine + melatonin + Zn‐Fe nano‐chelate treatments.

The longest fruits (3.50 cm) were observed in plants treated with T24 and T25, not significantly different from those treated with T9, T10, T20–T24, and T26–T28 (Figure 2). Spraying the apricot trees with arginine 150 ppm + melatonin 400 μmol/L + Nano‐chelate Zn‐Fe 2 g/L (T27) and 3 g/L (T28), respectively, caused increases in fruit length by 25.77% and 20.26% compared to control trees. The lowest fruit length (2.30 cm) was observed in plants treated with T11, which had no significant difference from the control (2.58 cm) and plants treated with T2–T6, T12, T14, and T16–T18 (Figure 3).

#### 3.2.5 Fruit Firmness

The firmest fruits (13.28 Ib/in^2^) were observed in plants treated with arginine 150 ppm + melatonin 400 μmol/L + Nano‐chelate Zn‐Fe 3 g/L (T28), which were not significantly different from those treated with T12–T14, T17, T23, T26, and T27 (Figure 2). Spraying trees with arginine 150 ppm + melatonin 400 μmol/L + Nano‐chelate Zn‐Fe 2 g/L and 3 g/L (T27 and T28) increased fruit firmness by 21.14% and 21.94%, respectively, compared to control. The lowest firmness (10.37 Ib/in^2^) was observed in the control trees, which were not significantly different from those treated with T2–T8 (Figure 3).

### 3.3 Biochemical Attributes

According to Figures 2 and 3, application of 150 mg/L arginine + 400 μM melatonin + 2 or 3 g/L iron‐zinc nano‐chelate significantly increased vitamin C, TSS, sugar content, and mineral elements (calcium, magnesium, potassium, zinc, and iron), while reducing fruit acidity.

#### 3.3.1 Total Soluble Solid

The highest TSS (14.10%) was recorded in plants treated with T25, not significantly different from trees which were treated with T20, T24, and T26–T27 (Figure 2). Application of arginine 150 ppm + melatonin 200 μmol/L + Nano‐chelate Zn‐Fe 3 g/L (T25) increased TSS by 18.91%, whereas T27 and T28 increased TSS by 17.60% and 17.72% compared to control. The lowest value (11.40%) was observed in plants treated with T3, not significantly different from control, and trees treated with T2 and T4 (Figure 3).

#### 3.3.2 Total Acidity (%)

**FIGURE 4:**
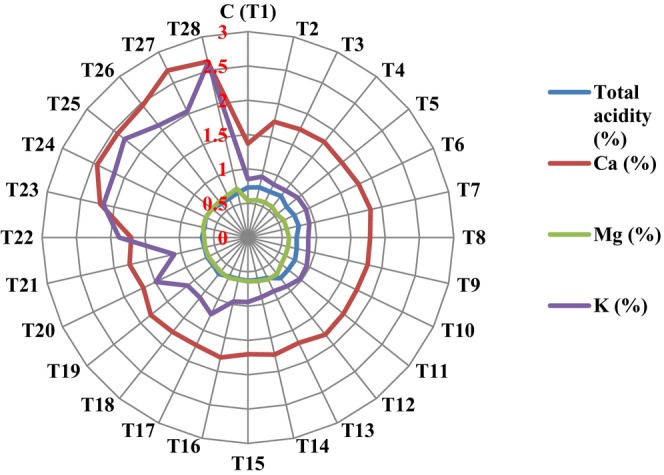
Means comparison (Duncan's Test: *p* < 0.05) total acidity (%), and the content (%) of Ca, Mg, and K in the apricot fruit under different arginine + melatonin + Zn‐Fe nano‐chelate treatments.

Treatments T16, T22, T27, and T28 had the most pronounced effect on reducing fruit acidity, decreasing it by 15.07%, 14.61%, 13.70%, and 8.63%, respectively, compared to the control (Figure 4). The lowest acidity (0.62%) was observed in fruits from T16‐treated trees, which did not differ significantly from T13 to T15, T17, T19, T21, and T23 (Figure 4). The highest acidity (0.77%) was recorded in T4, which was not significantly different from the control or treatments T2 to T3, T6 to T7, and T9 to T12.

#### 3.3.3 Vitamin C

The highest vitamin C content (15.60 and 15.53 mg per 100 g fresh weight) was recorded in fruits treated with T27 and T28, respectively, showing increases of 20.73% and 20.36% compared to the control. The lowest vitamin C content (12.03 mg/100 g FW) was observed in T3, which was not significantly different from the control and trees treated with T2 to T17 (except T15). Application of T27 and T28, respectively, increased the content of vitamin C by 20.73% and 20.39% compared to the control, respectively.

#### 3.3.4 Total Sugar

Treatments T2 to T4, T7, and T10 did not significantly affect total sugar content, whereas all other treatments led to significant increases. The highest sugar contents (11.3% and 11.4%) were observed in trees treated with T27 and T28, representing increases of 33.51% and 33.58%, respectively, compared to the control (Figure 3).

### 3.4 Mineral Content of Fruits

#### 3.4.1 Calcium (Ca)

The lowest calcium content (1.37%) was recorded in the control. Treatments T20 to T28 significantly increased fruit calcium levels, with the highest observed in T27 (2.70%), followed by T28 (2.63%). These two treatments increased calcium content by 49.26% and 47.84%, respectively, compared to the control (Figure 4).

#### 3.4.2 Magnesium (Mg)

The lowest magnesium content (0.537%) was found in trees treated with T5, which was not significantly different from the control and treatments T2 to T4. The highest magnesium content (0.733%) was recorded in fruits from T28‐treated trees, representing a 26.36% increase compared to the control (Figure 4). T27 increased Mg level by 20.97%.

#### 3.4.3 Potassium (K)

Treatments T2 to T16, T18 to T19, and T21 did not significantly affect potassium content. However, the other treatments significantly increased it compared to the control (Figure 4). The highest potassium level (2.607%) was recorded in fruits from trees treated with T28, which was not significantly different from those treated with T25 and T26. T27 and T28 increased potassium content by 58.40% and 67.43% compared to the control (Figure 4).

#### 3.4.4 Zinc (Zn)

**FIGURE 5:**
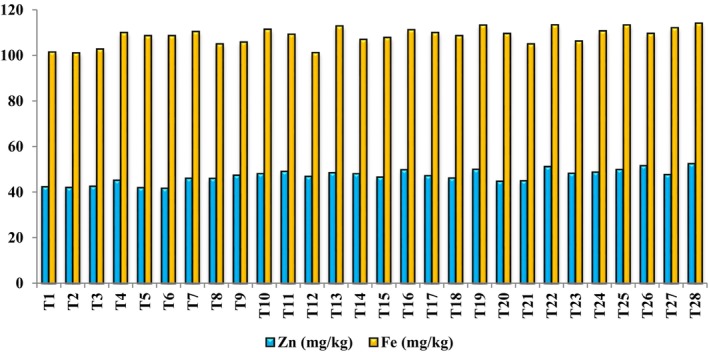
Means comparison (Duncan's Test: *p* < 0.05) the contents of Zn and Fe (mg/kg) in the apricot fruit under different arginine + melatonin + Zn‐Fe nano‐chelate treatments.

As presented in Figure 5, the highest fruit zinc content (52.43 mg/kg) was measured in fruits obtained from trees treated with T28, reflecting a 19.25% increase compared to the control. Treatment T26 had the next most significant effect on increasing zinc content (17.90%); however, T27 increased Zn content by 11.19% compared to the control. The lowest zinc content (41.70 mg/kg) was observed in response to T6, with no significant difference from the control and treatments T2, T3, and T5 (Figure 5).

#### 3.4.5 Iron (Fe)

Treatments T2, T3, and T12 had no significant effect on iron content. The highest iron content (114.10 mg/kg) was observed in trees treated with T28, followed by T24, T25, and T27 (Figure 5). The treatments of T27 and T28 increased Fe content by 9.46% and 11.07%, respectively, compared to the control, likely because of the higher concentration of Zn‐Fe Nano‐chelate.

### 3.5 Principal Component Analysis (PCA)

**FIGURE 6:**
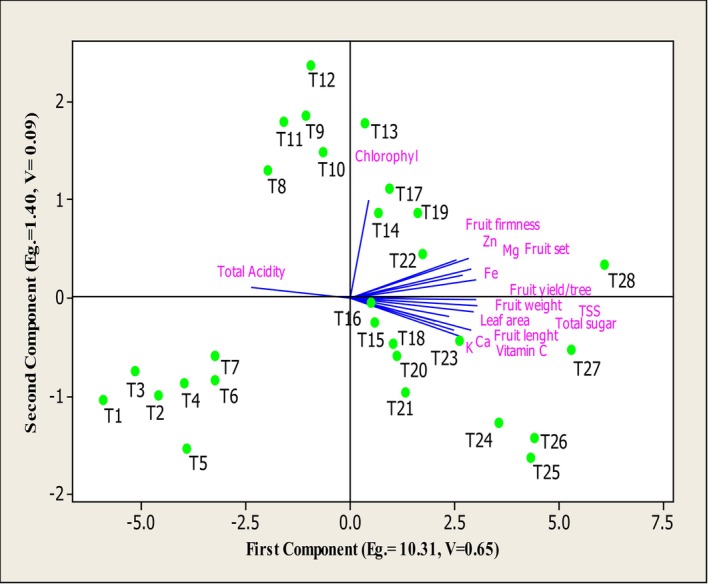
The biplot of first and second components for the studied traits on the basis of different treatments of arginine + melatonin + Zn‐Fe nano‐chelate.

Principal component analysis (PCA) also grouped the treatments into three categories. The first and second principal components had eigenvalues above 1 and together explained 74% of the total variance. Component 1 accounted for 65%, and Component 2 for 9% of total variance. Traits with the highest positive loadings in Component 1 were Fruit weight, Fruit yield/tree, Total sugar, TSS, Mg, Vitamin C, Fruit firmness, and Fruit set. Total acidity had the highest negative contribution. In Component 2, SPAD, Fruit firmness, Zn, and Mg contributed positively, whereas K, Vitamin C, and Ca had the most negative influence (Table 1). The biplot (Figure 6) revealed a strong correlation between treatments T27 and T28 (arginine (150 ppm) + melatonin (400 μmol/L) + Nano‐chelate Zn‐Fe (2 g/L) and 0.3 g/L) with key traits such as fruit yield, fruit firmness, fruit set, leaf area, TSS (total soluble solids), total sugars, vitamin C, calcium, magnesium, potassium, zinc, and iron. Total acidity (TA) was located in the second quadrants and had the strongest association with treatments T1 to T21. Overall, PCA showed that treatments T27 and T28 had the most positive impact on the qualitative and quantitative traits of apricot. These treatments caused significant increases in the following parameters: Fruit yield/tree, Fruit weight and length, Fruit firmness, Fruit set, Leaf area and chlorophyll index, TSS and total sugars, Vitamin C content, and mineral contents including calcium, magnesium, potassium, zinc, and iron. These treatments also caused a significant reduction in total acidity (TA), which contributes to improving the fruit's flavor quality of apricot.

**TABLE 1:** The share of variables in the first to fourth components.

Variable	PC1	PC2	PC3	PC4
Leaf area	0.228	−137	−0.069	−0.088
SPAD	0.044	0.709	−0.421	−0.145
Fruit set	0.259	0.169	0.049	0.269
Fruit yield/tree	0.291	0.135	0.218	−0.111
Fruit weight	0.293	−057	0.196	−0.054
Fruit length	0.197	−167	−0.477	−0.122
Fruit firmness	0.275	0.286	0.098	−0.137
TSS	0.286	−0.101	0.009	−0.196
Total acidity	−0.227	0.080	−0.487	0.106
Vitamin C	0.280	−234	−118	−0.035
Total sugar	0.290	−009	0.130	−0.195
Ca	0.239	−0.230	−0.364	0.070
Mg	0.281	0.206	0.026	−0.171
K	0.254	−0.274	−0.298	0.043
Zn	0.247	0.277	−0.012	0.351
Fe	0.190	0.005	0.040	0.776

T1‐T28: different levels of Arginine +Melatonin + Nano‐chealate Zn‐Fe, respectively including T1 = 0 ppm + 0 μmol/L + 0 g/L, T2 = 60 ppm + 100 μmol/L + 1 g/L, T3 = 60 ppm + 100 μmol/L + 2 g/L, T4 = 60 ppm + 100 μmol/L + 3 g/L, T5 = 60 ppm + 200 μmol/L + 1 g/L, T6 = 60 ppm + 200 μmol/L + 2 g/L, T7 = 60 ppm + 200 μmol/L + 3 g/L, T8 = 60 ppm + 400 μmol/L + 1 g/L, T9 = 60 ppm + 400 μmol/L + 2 g/L, T10 = 60 ppm + 400 μmol/L + 3 g/L, T11 = 100 ppm + 100 μmol/L + 1 g/L, T12 = 100 ppm + 100 μmol/L + 2 g/L, T13 = 100 ppm + 100 μmol/L + 3 g/L, T14 = 100 ppm + 200 μmol/L + 1 g/L, T15 = 100 ppm + 200 μmol/L + 2 g/L, T16 = 100 ppm + 200 μmol/L + 3 g/L, T17 = 100 ppm + 400 μmol/L + 1 g/L, T18 = 100 ppm + 400 μmol/L + 1 g/L, T19 = 100 ppm + 400 μmol/L + 3 g/L, T20 = 150 ppm + 100 μmol/L + 1 g/L, T21 = 150 ppm + 100 μmol/L + 2 g/L, T22 = 150 ppm + 100 μmol/L + 3 g/L, T23 = 150 ppm + 200 μmol/L + 1 g/L, T24 = 150 ppm + 200 μmol/L + 2 g/L, T25 = 150 ppm + 200 μmol/L + 3 g/L, T26 = 150 ppm + 400 μmol/L + 1 g/L, T27 = 150 ppm + 400 μmol/L + 2 g/L, T28 = 150 ppm + 400 μmol/L + 3 g/L.

### 3.6 Estimation of Pearson's Correlation

**FIGURE 7:**
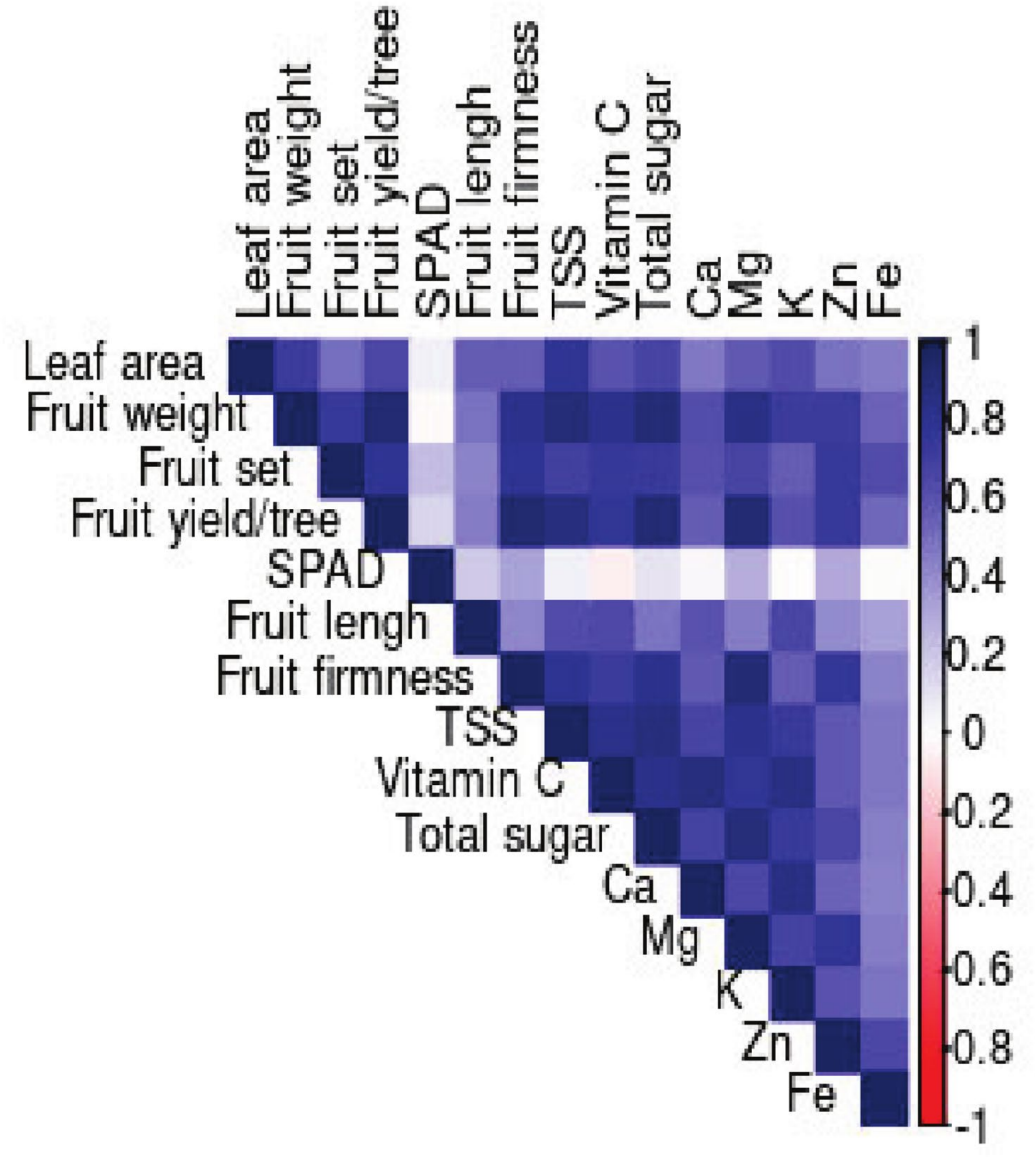
The heat map of Pearson's correlation between different traits under the effect of dissimilar levels of Arginine + Melatonin +Nano‐chealate Zn‐Fe.

The results of Pearson's correlation (Figure 7) revealed a significant positive correlation between leaf area and fruit weight (*r* = 0.73**) and TSS (*r* = 0.77**). The SPAD value has no positive or negative significant correlation with other variables. The fruit set showed a positive significant correlation with fruit yield/tree (*r* = 0.78**), fruit weight (*r* = 0.75**), the content of vitamin C (*r* = 0.74**), total sugar (*r* = 0.73**), and the content of Zn (0.75**). Fruit yield/tree displayed strong positive significant correlations with variables including fruit weight (*r* = 0.92**), fruit firmness (*r* = 0.93**), total sugar (*r* = 0.91**), and the content of Mg (*r* = 0.92**). In addition, fruit yield/tree has positive significant correlations with TSS (*r* = 0.87**), vitamin C (*r* = 0.77**), and the content of Zn (*r* = 0.74**); however, the correlation between fruit yield/tree and total acidity was negative and significant (*r* = −0.78**). Fruit weight has a significant positive correlation with fruit firmness (*r* = 0.82**), vitamin C (*r* = 0.82**), total sugar (*r* = 0.91**), Mg content (*r* = 0.83**), K (*r* = 0.73**), and Zn (*r* = 0.73**). Total acidity does not have a significant correlation with fruit length, Ca content, and SPAD value, whereas it showed negative significant correlations with all other variables. Fruit firmness showed a significant positive correlation with TSS (*r* = 0.79**), total fruit sugar (*r* = 0.82**), and Mg content (*r* = 0.92**). TSS had a significant positive correlation with the content of vitamin C (*r* = 0.83**), total sugar (*r* = 0.87**), Mg content (*r* = 0.80**), and potassium content (*r* = 0.74**). Vitamin C had a significant positive correlation with the traits total sugar (*r* = 0.82**) and nutrient content including Ca (*r* = 0.88**), Mg (*r* = 0.77**), and K (*r* = 0.86**). Total sugar had a significant positive correlation with Mg content (*r* = 0.88**) and potassium (*r* = 0.73**). The correlation between calcium content and potassium (*r* = 0.86**) and magnesium content with zinc (*r* = 0.76**) was positive and significant. Fruit iron content did not show a significant positive or negative correlation with any of the traits.

### 3.7 Regression Analysis

**FIGURE 8:**
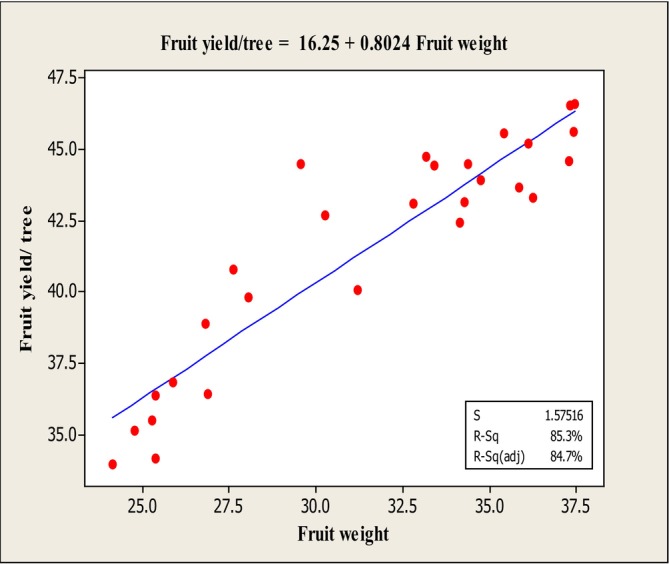
Diagram of linear regression between fruit yield/tree and fruit weight.

**FIGURE 9:**
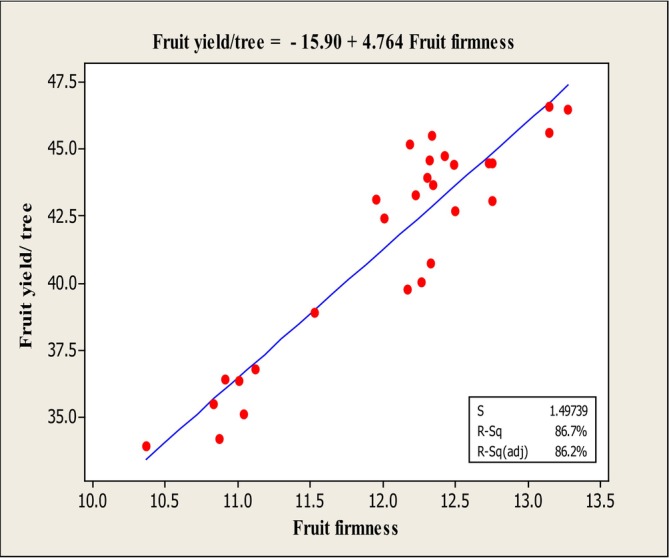
Diagram of linear regression between fruit yield/tree and fruit weight.

**FIGURE 10:**
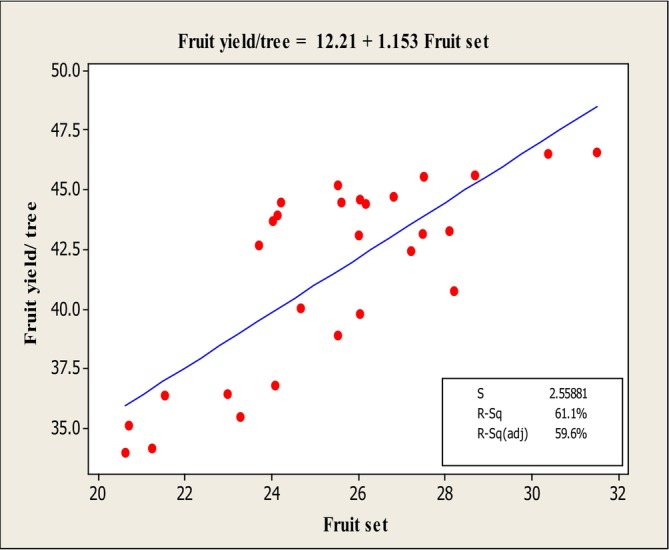
Diagram of linear regression between fruit yield/tree and fruit set.

The results of the stepwise regression analysis (Table 2) revealed that the traits fruit weight, fruit set, fruit firmness, and Mg content exhibited significant variance with fruit yield/tree at the 1% significance level. Consequently, linear regression analysis was performed between these traits and fruit yield/tree (Table 3), and the corresponding regression plots were generated (Figures 8, 9, 10).

**TABLE 2:** The summary of model in stepwise regression analysis.

Model	Model summary^b^			
	*R*	*R* ^2^	Adjusted *R* ^2^	Std. error of the estimate
	0.933^a^	0.87	0.862	1.5556

^a^ Predictors: (Constant), fruit weight, Mg, total acidity, fruit firmness, fruit set.

^b^ Dependent variable: fruit yield/tree.

**TABLE 3:** Results of ANOVA for linear regression between fruit yield/tree (dependent variable) and fruit weight, fruit set, fruit firmness, and Mg content as predictors (constant).

Model	df	Fruit weight	Fruit firmness	Fruit set	Mg content of fruit
Regression	1	267.54**	379.48**	267.54**	240.93**
Residual	26	6.548	2.24	6.55	7.57

** Significant at the 1% level.

**FIGURE 11:**
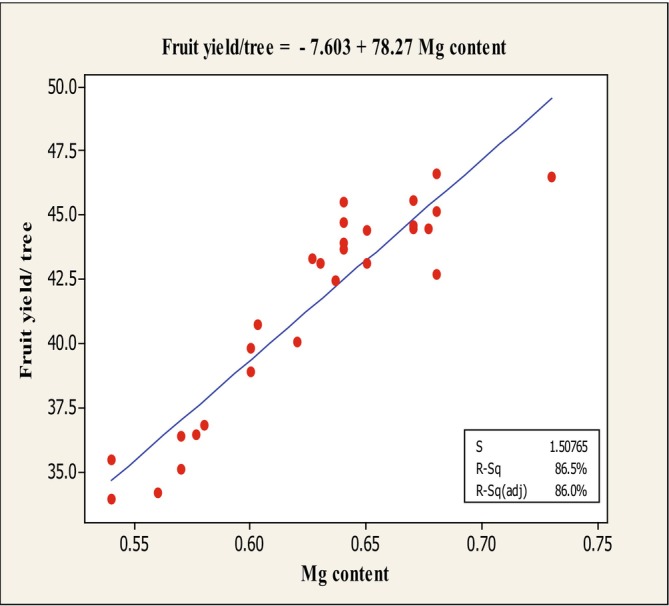
Diagram of linear regression between fruit yield/tree and Mg content.

The results of the individual linear regressions of these traits on fruit yield/tree (Table 3) are as follows:

The linear regression analysis between fruit yield/tree and fruit weight (Figure 8) indicated a positive linear relationship observed between the independent variable (fruit weight) and the dependent variable (fruit yield). The adjusted coefficient of determination (*R* ^2^ adj) for this regression was 84.7%, and the fitted linear equation was: *Y* = 16.25 + 0.802*X*. The linear regression between fruit yield/tree and fruit firmness (Figure 9) revealed a negative linear relationship between the independent variable (fruit firmness) and the dependent variable (fruit yield/tree). This suggests an inverse association between fruit firmness and yield. The adjusted *R* ^2^ for this model was 86.2%, and the fitted linear equation was: *Y* = −15.90 + 4.724*X*. According to Figure 10, there was a positive linear relationship between the independent variable (fruit set) and the dependent variable (fruit yield/tree), indicating that fruit yield increases with higher fruit set. The adjusted *R* ^2^ for this regression was 59.60%, and the fitted linear equation was: *Y* = 12.21 + 1.153*X*. Finally, the linear regression between fruit yield/tree and Mg content showed an inverse relationship, suggesting that higher Mg content is associated with reduced yield (Figure 11). The adjusted *R* ^2^ for this regression was 86.00%, and the fitted linear equation was: *Y* = −7.603 + 27.78*X* (Figure 11).

## 4 Discussion

In this study, the combination of foliar application of arginine, melatonin, and Zn‐Fe nano‐chelate significantly increased the yield of apricot, fruit firmness, fruit size, and several nutraceutical or nutritional traits such as Vitamin C, total soluble solids (TSS), sugars, and mineral contents (Ca, Mg, K, Zn, and Fe). This synergistic improvement indicates that the compounds activate various complementary physiological and biochemical responses to improve fruit quality and productivity. Total acidity also decreased, which will improve consumer acceptance and store life. Zn and Fe nano‐chelates consist of micronutrients that have high bioavailability that facilitate physiological mechanisms of accumulation in fruits. Nanoparticles boost the efficiency of nutrient uptake given their high ratio of surface area to volume and their reactivity. For example, previous studies of horticultural crops including strawberries and tomatoes have demonstrated that foliar application of Zn‐Fe nano‐chelates boosted total yield and metabolic content of foods (Fatemi et al. 2020). In a recent study with apple trees, the researchers reported that fruit biomass increased with treatment of fruit with nano‐chelated micronutrients (Rahman et al. 2025). Treatment with nano‐chelates enhanced the vitamin and polyphenol content in apples. The nano‐chelates act to promote enzyme activity, photosynthesis, and the biosynthesis of metabolites related to quality.

Application of arginine + melatonin Zn‐Fe nano‐chelate resulted in the improved in the apricot fruit yield; fruit physical properties: firmness, weight, and length; and chemical attributes: the contents of vitamin C, soluble sugars, total soluble solids, the concentrations of calcium, magnesium, potassium, zinc, and iron. Our results seem to be related to the synergistic effect of these compounds. In addition, the use of combined treatments was effective in reducing total acidity, which can improve the taste and postharvest life of the apricot fruit. Although there has been no report on the combined use of melatonin, arginine, and zinc‐iron nano‐chelate on the qualitative and quantitative performance of fruits, they solely applied. Melatonin is a multifunctional regulatory biomolecule with pleiotropic effects in the plants (Altaf, Shahid, Ren, Altaf, et al. 2021). It delays senescence and promoted photosynthesis, root development, and fruit maturation (Altaf, Shahid, Ren, Mora‐Poblete, et al. 2021; Jahan et al. 2021; Nawaz et al. 2016). In strawberry, exogenous melatonin (0.1 mmol L^−1^) has enhanced the expression of FaTDC, FaT5H, FaSNAT, and FaASMT genes involved in MT biosynthesis, resulting amplified the endogenous melatonin levels (Liu et al. 2018). Spraying at 100 μmol L^−1^ Melatonin on summer black grape induced transcript abundance of anthocyanin biosynthesis‐related genes, lead to skin coloration (Xia et al. 2021). The exogenous melatonin application has been associated with increased fruit yield through restoring chlorophyll content, root architecture, and gas exchange parameters as observed in tomato (Altaf et al. 2022). Previous studies have shown that 100 μM melatonin enhances growth and productivity in tomato plants, leading to higher fruit yields (Ibrahim et al. 2020; Liu et al. 2016). Melatonin maintains fruit firmness and quality across various fruits, such as mangoes and peaches (Rastegar et al. 2020; Wu et al. 2023). Postharvest treatment with the 100 μmol·L^−1^ MT has been found to improve the physical properties of sweet cherries fruits, by increasing fruit firmness and TSS (Wang et al. 2019) and tomato (Ibrahim et al. 2020). Postharvest application of 0.1 or 1 mmol L^−1^ of melatonin maintains color, firmness, and the total soluble solids content of strawberry fruit (Liu et al. 2018). The application of melatonin encourages the buildup of secondary and primary metabolites, resulting significantly enhance the chemical attributes such as vitamin C, sugars, amino acids, and proteins (Wang et al. 2019). Additionally, melatonin enhances the accumulation of volatile substances, phenolic acids, and flavonoids, contributing to improved flavor quality and nutritional (Dou et al. 2022) 100 μmol L^−1^ Melatonin has increased the soluble sugar content in black grape by promoting the activity of sucrose phosphate synthase (Xia et al. 2021). In addition, it improved the content of N, K, Cu, Fe, and Zn in grape berries (Xia et al. 2021). MT‐treated tomato showed improving ascorbic acid and lycopene (Ibrahim et al. 2020). Additionally, melatonin treatment boosts the concentration of essential minerals like calcium, magnesium, potassium, zinc, and iron in fruits (Dou et al. 2022; Ibrahim et al. 2020; Liu et al. 2016). Melatonin enhances the activity of antioxidant enzymes, which helps in reducing oxidative damage in plants. This activity is crucial for maintaining fruit quality by reducing the levels of harmful substances such as hydrogen peroxide and malondialdehyde (Ibrahim et al. 2020; Liu et al. 2016). Although the beneficial effects of melatonin on fruit quality and yield are evident, further research is needed to understand the molecular mechanisms underlying these effects. This knowledge could aid in developing strategies for quality improvement breeding (Dou et al. 2022). Also, the application of melatonin in agricultural practices requires optimization to maximize its benefits under various environmental conditions. Future studies should focus on determining the optimal concentrations and application methods for different crops and growing conditions (Dou et al. 2022; Ibrahim et al. 2020; Liu et al. 2016; Wang et al. 2019). Arginine, a multi‐functionality amino acids, is a precursor of polyamines biosynthesis and signaling molecules such as nitric oxide (Fung et al. 2025; Kurhaluk and Tkaczenko 2025). It has been reported that arginine application increases the expression of the LeNRT1.1 gene and consequently increases root activity and nitrogen uptake and transport, which successively leads to an increase in the photosynthetic rate and plant growth (Wang et al. 2021). Strawberry plants treated by 250 and 500 μM arginine presented increased fruit weight and yield (Mohseni et al. 2017). Arginine improved fruit quality, that is, in tomato fruits, it has improved the content of vitamin C, soluble solid, soluble sugar, and titratable acids (Wang et al. 2021), and in strawberry 1 mM Arg maintains fruit quality, indicated by firmness, titratable acid, soluble solid content, vitamin C, anthocyanin and total phenolic content (Shu et al. 2020). Exogenous application of Arg has improved fruit weight, firmness, and soluble solid content of blueberries (Wang, Wang, et al. 2023). Sweet cherry, treated with 400 μM arginine in the postharvest, showed the increased titratable acid, soluble solids, and vitamin C, as well the firmness of fruits (Pakkish and Mohammadrezakhani 2022). 250 and 500 μM arginine has increased total soluble solid, sugar, titratable acidity, anthocyanin, phenol, and vitamin C content in strawberry (Mohseni et al. 2017).

In a pre‐harvest experiment, application of 500 or 1000 ppm glycine, arginine, and glutamic acid, solely or in combination, increased leaf chlorophyll, fruit set, fruit yield, fruit firmness, total soluble solids, vitamin C, and total sugars, as well as the content of N, K, and P in the leaf of Guava Trees (Almutairi et al. 2022). On the basis of a transcriptome data, Arg changes transcripts of the genes involved in fruit firmness, anthocyanin content, sugar content, indole‐acetic acid (IAA) content, abscisic acid (ABA) content, and ethylene emissions (Lv et al. 2020). Arginine is a precursor of polyamines biosynthesis and signaling molecules such as nitric oxide. In strawberry fruit, application of 1 mM Arg triggered NO accumulation, resulting from higher NOS activity, which is associated with a higher vitamin C, anthocyanin, and total phenolic content, and the activities of the antioxidant enzymes (Shu et al. 2020). Also, the studies revealed that the activities of the defense enzymes—phenylalanine ammonialyase (PAL), chitinase (CHI), β‐1,3‐glucanase (GLU) and polyphenol oxidase (PPO) induced with exogenic Arg application (Shu et al. 2020).

The availability of macro and micronutrients is essential for plant growth and improving fruit quality. Zn and Fe are involved essential elements in biochemical and physiological plant processes (Fatemi et al. 2020; Guardiola‐Márquez et al. 2023). The nano‐chelated Zn and Fe formulations enhance the absorption efficiency of micronutrients, contributing to higher levels of minerals in the plant and better physiological function. For example, in soybean plants, foliar application of Fe‐Zn nano‐chelates caused increases in the seed weight and yield, leaf area, and chlorophyll concentration (Vaghar et al. 2020), and in apple trees, the Zn and Se nanoparticles have enhanced the fruit yield and bioactivecompounds in the apple fruits (Montaño‐Herrera et al. 2022). 200 μg g^−1^ nano‐Zn significantly increased fruit set (16.9%), yield (48.3%), and metabolic content of strawberry (Saini et al. 2021). Spraying peach trees with Zn nanoparticles strongly enhanced growth indicators such as shoot diameter, leaf area, total chlorophyll, and percentage of flowers, thereby enhancing yield. Additionally, the fertilizer also enhanced fruit weight, firmness, diameter, length, size, soluble solids, and sugars content. Similarly, there was an improved concentration of anthocyanin and vitamin C content, whereas the fruit acidity was significantly less than the control (Mosa et al. 2021). Spraying strawberries with borax at 0.6% increased the percentage fruit set, fruit retention percentage, specific gravity, fruit length, weight, volume, pulp weight, total sugar, TSS, fruit yield, and fruit content while decreasing the fruit drop percentage (Tiwari et al. 2023). Both rates of 500 and 1000 ppm of ZnO NPs and 1000 ppm of B2O3 NPs had strong positive and significant increases in chlorophyll concentration of leaves, fruit set percentage, and productivity; all leaf nutrient content potassium, phosphorous, calcium, nitrogen, boron, and zinc also decreased fruit drop percentages (Abd El‐wahed et al. 2024). A nano‐chelate compound containing 4% urea, 3% iron, 2% manganese, and 1% boron has increased yield/plant, total soluble solids, and titratable acid of grapevines (Arji et al. 2022). Also, 10 and 20 mg/L of Fe nano‐chelate have improved vitamin C (34%), total carotene (25%), flavonoid (17%), and polyphenol content (66%) in tomato (Rahman et al. 2023). The application of melatonin, in combination with arginine and Zn‐Fe nano‐chelate, shows promise in enhancing fruit yield and quality. The synergistic effect of arginine and melatonin in inducing endogenous polyamines, hormones (IAA), and ethylene emissions (Lv et al. 2020), as well as mineral availability (Zn, Fe, and N) regulates physiological activities and improves plant growth and development. Also, the results showed that ZnONPs enhanced the growth, chlorophyll content, and yield of wheat. These same effects were maximized in combination with MT (Chen et al. 2023). These results highlight the potential increase in crop benefits associated with the combination of melatonin and zinc, which is similar to our results in mung bean plants. Zhang et al. (2024) showed that melatonin improved the growth and waterlogging tolerance of cotton plants.

## 5 Conclusion

Although most of the applied treatments enhanced both the quantity and quality of apricot fruit, the highest levels of calcium, magnesium, potassium, zinc, iron, total sugar, and vitamin C were observed in plants treated with arginine (150 ppm) + melatonin (400 μmol/L) + Nano‐chelated Zn‐Fe at either 2 g/L or 3 g/L. These two treatments (T27 and T28) also resulted in the highest fruit yield, total soluble solids (TSS), fruit set, and firmness, along with reduced acidity. On the basis of a comprehensive evaluation of physiological, qualitative, quantitative, and nutritional traits of apricot fruits, the T27 and T28 treatment combinations are recommended as the most effective for enhancing yield and fruit quality. These combinations not only significantly increased fruit yield but also played a crucial role in improving key quality attributes, including firmness, sweetness, reduced acidity, and higher concentrations of essential vitamins and minerals. Therefore, they are proposed as the final recommendation for optimizing apricot production and fruit quality. The application of melatonin in combination with other compounds like arginine and Zn‐Fe Nano‐chelate shows promise in enhancing fruit yield and quality. Its role in improving physical and chemical attributes, along with its antioxidant properties, makes it a valuable tool in horticulture practices. Further research is needed to fully harness its potential and understand the underlying mechanisms.

## Author Contributions

**Raghad Adnan Ali AL‐Qady:** conceptualization (equal), methodology (equal). **Wasan Waleed Ahmad:** data curation (equal), funding acquisition (equal), methodology (equal). **Waad S. Faizy:** conceptualization (equal), methodology (equal), writing – original draft (equal). **Mustafa Natheer Mustafa:** conceptualization (equal), funding acquisition (equal), investigation (equal), methodology (equal). **Borzou Yousefi:** data curation (equal), funding acquisition (equal), investigation (equal), methodology (equal).

## Conflicts of Interest

The authors declare no conflicts of interest.

## Data Availability

All data generated during this study are included in the article.
